# Hepatitis B Virus Pregenomic RNA Reflecting Viral Replication in Distal Non-tumor Tissues as a Determinant of the Stemness and Recurrence of Hepatocellular Carcinoma

**DOI:** 10.3389/fmicb.2022.830741

**Published:** 2022-04-07

**Authors:** Yiwei Xiao, Junning Cao, Ze Zhang, Chaoting Zeng, Guomin Ou, Jihang Shi, Zhixiu Liu, Yi Li, Juan Deng, Yinzhe Xu, Wenwen Zhang, Jie Li, Tong Li, Hui Zhuang, Shichun Lu, Kuanhui Xiang

**Affiliations:** ^1^Department of Microbiology and Infectious Disease Center, School of Basic Medical Sciences, Peking University Health Science Center, Beijing, China; ^2^Faculty of Hepato-Pancreato-Biliary Surgery, Chinese PLA Genera Hospital, Beijing, China; ^3^Division of Pathology and Laboratory Medicine, Hebei Yanda Lu Daopei Hospital, Langfang, China; ^4^Peking University-YHLO Joint Laboratory for Molecular Diagnostic of Infectious Disease, Peking University, Beijing, China

**Keywords:** hepatitis B virus, viral replication, pgRNA, hepatocellular carcinoma, cancer stem cell

## Abstract

**Background:**

The existence of hepatic cancer stem cells (CSCs) contributes to chemotherapy resistance and cancer recurrence after treatment or surgery. However, very little is known about the hepatitis B virus (HBV) replication and its relationship with the stemness of hepatocellular carcinoma (HCC) in HBV-related HCC patients.

**Methods:**

We collected tumor tissues (T), matched adjacent non-tumor tissues (NT), and distal non-tumor tissues (FNT) from 55 HCC patients for analysis.

**Results:**

We found HBV DNA levels were higher in T samples than NT and FNT samples, but HBV pgRNA and total RNA expressed lower in T samples. HBV pgRNA and total RNA correlate to HBV DNA among the T, NT, and FNT samples. Further evidence for HBV replication in T samples was provided by HBV S, reverse transcriptase, and X genes sequencing, showing that HBV sequences and genotypes differed between T and matched NT and FNT samples. HBV pgRNA and total RNA showed more frequent significant correlations with CSC markers in NT samples in HBsAg-positive patients. The markers CD133 and OCT4 expressed higher in FNT samples, and HBV replication marker of pgRNA levels was significantly positively correlated to these two markers only in FNT samples. The detection of pgRNA and OCT4 in FNT was correlated to the recurrence of HCC in the resection of HCC patients. Analysis of HBV receptor, sodium taurocholate co-transporting polypeptide (NTCP), showed that NTCP was correlated negatively to CSC markers in T samples, except for the CD44.

**Conclusion:**

HBV replication may present in HCC with a weak transcriptomic signature. Moreover, the expression level of HBV pgRNA in distal non-tumor tissues is a sensitive marker for HBV replication and prognosis, which is associated with CSC-related markers especially with OCT4 in distal non-tumor tissues and recurrence of HCC in HBV-related HCC patients.

## Introduction

Hepatocellular carcinoma (HCC) is one of the most common causes of cancer death worldwide in the past decades (GBD 2017 Causes of Death Collaborators, 2018). Despite the availability of hepatitis B virus (HBV) vaccine, 257 million people with existing chronic HBV infection and the children infected by mother-to-child transmission or horizontal transmission in early childhood are still at high risk for developing liver cancer (Polaris Observatory, 2018). It is reported that about 50% of HCC cases are attributed to persistent HBV infection (Parkin et al., 2001; Montalto et al., 2002).

Hepatitis B virus is a non-cytopathic and partially double-stranded hepatotropic DNA virus. In the nucleus of hepatocytes, the relaxed circular double-stranded HBV DNA (rcDNA) is repaired into covalently closed circular DNA (cccDNA), which is the template for viral RNA transcription. HBV pgRNA is reverse-transcribed to the synthesis of HBV DNA; 2.4/2.1-kb mRNAs are translated to hepatitis B surface antigen (HBsAg) and 0.7-kb RNA is translated to hepatitis X protein (HBx) (Mani and Andrisani, 2018).

The cancer stem cells (CSCs) theory suggests that tumors are composed by heterogeneous cells with different grade of differentiation (Toh et al., 2017). The cells exhibit characteristics including extensive self-renewal, expression of pluripotency genes, altered expression of genes involved in cellular metabolism, cell cycle progression, and potent tumor initiating (Safa, 2016; Mancini et al., 2018). Similarly, CSCs have been found in several cancers, such as liver cancer and colon cancer (Al-Hajj et al., 2003; O’Brien et al., 2007; Arzumanyan et al., 2011; Anfuso et al., 2018). Accumulating evidence supports the CSCs existing in the cancer, which contribute to chemotherapy resistance and cancer recurrence after treatment or surgery (Widschwendter et al., 2007). Epithelial cell adhesion molecule (EPCAM), CD133, CD44, SOX2, and octamer-binding transcription factor 4 (OCT4) are well-known as liver CSC markers in human HCC and contribute to the stemness of cancer cells (Mani and Andrisani, 2018). Therefore, the new potential treatment target of cancer is the CSCs.

Since HBV infection and replication requires the receptor of sodium taurocholate co-transporting polypeptide (NTCP) and transcription factor HNF4α expressed in differentiated hepatocytes, there is still a controversy if HBV can replicate in tumor tissues (T) of HCC patients (Yan et al., 2012; Park et al., 2020). Many studies suggested that tumor cells do not support HBV replication, where they only contain integrated viral DNA. The integrated DNA sequences fragmented in the cells cannot support HBV replication and transcription of pgRNA (Wang et al., 1991). However, cccDNA and pgRNA may be present in HCC cells indicating HBV replication in tumor cells. Halgand et al. (2018) reported that several evidences supported that HBV could replicate in tumor cells, which showed pgRNA and cccDNA representing a sensor for viral replication and prognosis.

Hepatitis B virus replication may be involved in the contribution of CSC formation. For example, Liu et al. reported that HBV preS1 protein was required for HBV-mediated CSC generation. PreS1 protein could activate CD133, CD117, and CD90 expression in normal hepatocyte derived cell line (LO2) and human hepatoma cell line (HepG2 and Huh7) (Liu et al., 2017). Mani et al. (2016) reported that EPCAM might be an hCSC-like gene signature in HBV associated HCCs. In addition, the expression of CD133, CD34, and alpha fetal protein (AFP) rather than EPCAM were significantly higher in HCC compared to paired non-HCC tissue, indicating the activation of hepatic stem cells compartment during hepatocellular carcinogenosis in the HBV-transgenic mouse model (Anfuso et al., 2018). However, the overall analysis on the relationship of HBV replication and CSCs in HCC patients has not yet been well-studied.

Here, we analyzed the viral and cellular parameters in paired tissues of 55 HCC patients to determine the relationship of replicating HBV and CSCs.

## Materials and Methods

### Study Patients

The 55 HCC patients were enrolled from 2017 to 2018 in the First Medical Center of Chinese PLA General Hospital, Beijing, China. Of these, nine had HCC of unknown causes (cryptogenic HCC), and 46 had HCC with chronic hepatitis B (CHB). Anti-HBc antibody was detectable in five out of nine cryptogenic HCC patients; anti-HBs were detectable in other patients. We collected the frozen samples of HCC and matched liver tissues [adjacent non-tumor (NT): <3 cm from the outer edge of tumor, distal non-tumor (FNT): >3 cm from the outer edge of tumor] just after tumor resection combined with relative clinical information. Informed consent was obtained regarding the use of their samples for further studies. All patients had no serological markers of hepatitis C virus, hepatitis D virus, and human immunodeficiency virus infection.

The baseline characteristics of 46 CHB-related HCC and nine cryptogenic HCC patients are shown in Table 1. Histological analysis showed that 46 cases were CHB-caused HCC. Tumor size was defined by its largest dimension. Fifteen patients received antiviral treatment with nucleos(t)ide analogues. Viremia was detectable in 35 patients. Four CHB related HCC patients were lost to follow-up.

**TABLE 1:** Baseline characteristics of 46 HBsAg-positive and 9 HBsAg-negative patients.

Variables	CHB related HCC	Cryptogenic HCC
Number	46	9
Age (years)	52.87 ± 10.40 (14–74)	57.29 ± 14.19 (28–72)
Male/Female (count)	42/4	6/3
HBV history (years)	17.13 ± 8.64 (2–30)	/
AFP serum (μg/L)	2853 ± 6036 (2.1–24200)	9.94 ± 19.31(1.25–53.60)
ALT (median, range, U/L)	32.55 (13.10–472)	19.70 (7.3–163.3)
AST (median, range, U/L)	30.20 (12.40–646)	18.30 (10.40–61.00)
HBeAg positive (count)	12	/
HBV DNA (serum)	35/46	/
>10000 IU/ml	15	
1000–10000 IU/ml	2	
100–1000 IU/ml	10	
20–100 IU/ml	3	
<20 IU/ml	5	
Not detected	11	
Antiviral therapy (AVT)	15/46	/
Adefovir	3	
Lamivudine + Entecavir	1	
Entecavir	7	
Tenofovir	1	
IFN-α	1	
Unknown AVT	2	
Largest tumor size (cm) (median, range)	5.25 (1.2–19)	
Vascular invasion (count)	21/46	
Edmondson score		
I/II (High)	7	
III (Middle)	34	
IV (Low)	5	
Follow-up (days) (mean)	604.12 ± 95.54	388.3 ± 228.2
HCC recurrence/death (count)	19/2	4
Lost to follow-up	4	2

*AFP, alpha fetoprotein. Normal distribution data were expressed as mean ± standard deviation. Non-normal distributions data were expressed as median and range.*

### Preparation of DNA and RNA

Total DNA was extracted with the DNeasy Blood & Tissue Kit (QIAGEN, Germany) from liver tissues after homogenization of the frozen tissues with precellys beads. Total RNA was extracted with TRIzol (Invitrogen, Carlsbad, CA, United States) and treatment with DNase. DNA and RNA were quantified using Nanodrop.

### Quantification of Hepatitis B Virus DNA, cccDNA, pgRNA, Total RNA, and mRNA of Cancer Stem Cell-Related Markers

The qPCR for HBV DNA quantification was performed as previously described (Michailidis et al., 2017; Xiang K. H. et al., 2019). Briefly, serum HBV DNA was detected by the COBAS^®^ AmpliPrep/COBAS^®^ TaqMan^®^ HBV test version 2.0 (Roche, Switzerland). HBV DNA from liver tissue was quantitated by qPCR with TaqMan Universal PCR Master Mix (Applied Biosystems, Foster City, CA, United States). The PCR was performed in a 5-μl reaction volume as described in manual protocol. The primers were shown in Supplementary Table 1. The protocol for PCR on HBV DNA was as follows: 50°C for 2 min, 95°C for 10 min then 40 cycles of 95°C for 15 s, and 60°C for 1 min.

For HBV cccDNA quantification, the DNA samples were treated with Plasmid-safe DNase to degrade the cellular linear DNA form. In order to confirm the efficient treatment, the β-globin copy number decreased > 100-fold, indicating the effectiveness of DNase treatment (Halgand et al., 2018). The qPCR was performed in a 5-μl reaction volume as described in manual protocol. The protocol for qPCR on cccDNA was as follows: 50°C for 2 min, 95°C for 2 min then 40 cycles of 95°C for 15 s, 60°C for 15 s, and 72°C 1 min.

Quantification of HBV total RNA, pgRNA, HBV receptor NTCP, HCC clinical marker AFP, and CSC-related markers including EPCAM, CD133, CD44, SOX2, and OCT4 were described before (Xiang et al., 2017). In brief, HBV total RNA was extracted and reverse-transcripted into cDNA by SuperScript III RT kit (Invitrogen). The cDNA of these markers was quantitated and normalized by ribosomal protein S11 (RPS11) gene. The qPCR was performed in a 5-μl reaction volume as described in manual protocol. The protocol for relative quantification PCR on all markers was as follows: 50°C for 2 min, 95°C for 2 min then 40 cycles of 95°C for 3 s, and 60°C for 30 s.

### Sequencing of Hepatitis B Virus RT, X, and Integrated RT Fragment Sequences Amplified by Inverse PCR in Tissue Samples

Hepatitis B virus sequences X and RT regions were amplified from tissues samples and sequenced as described previously (Xiang et al., 2018). HBV S gene was obtained from RT due to its overlap with RT region. PCR was performed in a 50-μl reaction volume. The protocol for amplifying PCR on X region was as follows: 30 cycles of 98°C for 10 s, 60°C for 30 s, and 72°C for 1 min.

Inverse PCR was used to detect integration junctions between the RT region of HBV double-stranded linear DNA and host DNA. We designed primers for the inverse PCRs and sequenced the HBV sequences between nt 246 and 680. The protocol of inverse PCR was followed by others with little modification by introducing the new restriction enzyme cleavage sites of *Xba*I, *Spe*I, and *Xma*JI (Mason et al., 2010). Briefly, total DNA isolated from 1-mg liver fragments was cleaved by *Xba*I to detect integration junctions near this location in viral DNA. The cut DNA was circularized by incubation with T4 DNA ligase and was cut with the *Spe*I restriction endonuclease to produce linear molecules in which virus-cell junctions are flanked by viral sequences. These fragments were serially diluted into 96-well PCR trays and were amplified by nested PCR using the primers described in Supplementary Table 1. Then, the products of nested PCRs were subjected to electrophoresis in 1.3% agarose gels and gel extraction. The gel-extracted products were sequenced with the appropriate forward primer that map from nt 246 and nt 680. Sequence alignments with HBV DNA and flanking DNA sequences on the human genome was determined using BLAST to search the “reference genomic sequences” in the database. For the real-time quantitative PCR, there were three PCR replicate wells per gene for the qPCR analysis; for inverse PCR and HBV sequences amplified, two replicate wells to test for successful amplification in a reaction.

### Histology and Immunostaining of Liver T, NT, and FNT Samples

A modified nuclear grading scheme outlined by Edmondson and Steiner was used for scoring tumor grades. Grades I and II were defined as high differentiated, grade III as moderately differentiated, and grade IV as lower differentiated. Microvascular invasion was determined in all cases by clinical detection.

In order to eliminate batch effects between the same samples, the tumor and adjacent non-tumor tissue from same patients were embedded in a single paraffin wax to make slides and perform IHC analysis. To observe the tumor tissues and differentiation, all liver tissues were collected for HE staining. For the immunostaining of fixed sections, we performed the immunohistochemical (IHC) analysis as described previously (Xiang C. et al., 2019). The primary antibodies for HBV and CSC-related markers hepatitis B core antigen (HBcAg), CD133, CD44, OCT4, and NTCP were anti-HBc (Dako, Glostrup, Denmark), anti-CD133 (Abcam, Cambridge, MA, United States), anti-CD44 (Servicebio, Wuhan, China), anti-OCT4 (Proteintech, United States), anti-NTCP (Sigma, St, Louis, MO, United States).

Panoramic scanning of slides was performed by using NanoZoomer Digital Pathology. Histochemistry score (H-score) was scored by QuPath software. For EPCAM, CD133, CD44, and NTCP, which were expressed on membranes, we scored them by calculating the DAB OD mean in cytoplasm. For OCT4, which were expressed in nucleus, we scored them by calculating the DAB OD mean in nucleus. For HBcAg, we scored them by calculating the DAB OD mean in cell (including cytoplasm and nucleus). Analyses of H-scores among T, NT, and FNT were compared by paired *t*-test.

### Southern Blot of Hepatitis B Virus DNA and Northern Blot of Hepatitis B Virus RNA

Southern blot was performed with the DIG High Prime DNA Labeling and Detection Starter Kit II (Roche, Basel, Switzerland). DNA sample (20 μg) was separated on a 0.8% agarose gel and then transferred to a nylon membrane (Amersham, Piscataway, NJ, United States). Blots were hybridized with a DIG-labeled DNA probe that covered the entire X gene of HBV.

Northern blot analysis total RNA. RNA samples (30 μg) were resolved on 1% formaldehyde–agarose gel and transferred to a Hybond N + nylon membrane. Northern bolt was performed with the DIG High Prime DNA Labeling and Detection Starter Kit II (Roche). Blots were hybridized with probe targets the full-length 3.2-kb HBV DNA probes.

### Public Data Analysis

We used UCSC Xena^1^ to perform survival analysis of OCT4 in the data HBV antigen annotated samples from TCGA Liver Cancer project.

### Statistical Analysis

Statistical analysis was performed by using the SPSS software and R software. Categorical variables were expressed as % (m/n) and examined by chi-square/Fisher’s exact test. Normal distributions data were expressed as mean ± standard deviation and compared by the Student’s *t*-test. Non-normal distribution data were expressed as median and interquartile range (IQR) or (range) and compared by Mann–Whitney *U* test. Matched or paired data were compared by Wilcoxon signed rank test. Non-parametric Spearman and binary logistic regression models were used when appropriate. Logistic regression was performed to develop the predictive model.

For survival analysis, continuous variables were performed by ROC curve analyses to find the optimal cut-off value for conversion into binary categorical variables. Log-rank test and Kaplan–Meier plots were used to identified variables and describe survival rates. All *p*-values were two-tailed and a *p*-value < 0.05 was considered significant.

## Results

### Comparison of Hepatitis B Virus Markers Among T, NT, and FNT Compartments

**FIGURE 1:**
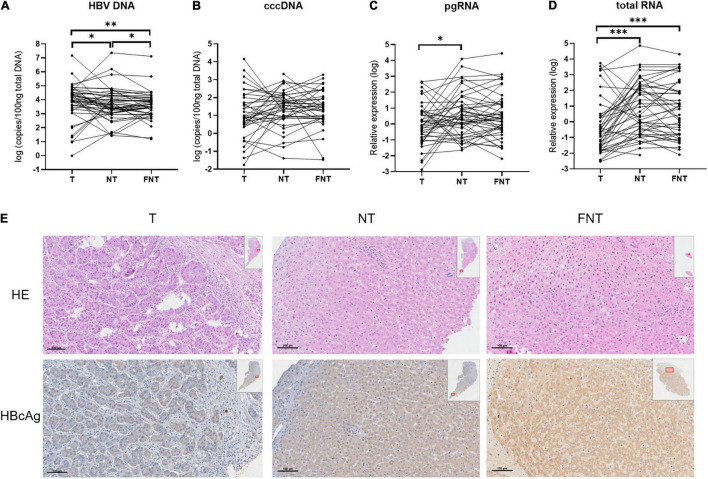
Comparisons of HBV markers among T, NT, and FNT compartments. Dot plots of **(A)** HBV DNA, **(B)** cccDNA, **(C)** pgRNA, and **(D)** total RNA levels. **(E)** Immunohistochemical analysis of HBcAg expression from the same patient. Statistical analysis was performed by Wilcoxon signed rank test. *p < 0.05; ^**^p < 0.01; ^***^p < 0.001. T, tumor; NT, adjacent non-tumor; FNT, distal non-tumor. Lines of histogram plots means average values.

We performed quantitative detection of intrahepatic HBV DNA in all tissues of 55 HCC patients. The HBV DNA was all positive in all the CHB-related HCC. Of 27 tissues from nine cryptogenic HCC patients, HBV DNA was detectable in all patients and 22 of 27 tissues (>10 copies/100 ng). Given the detection of DNA, it is therefore likely that these patients had OBI. We note that up to four of nine of these cryptogenic HCC patients experienced recurrence or death, possibly implying an association with reactivation of OBI (Wong et al., 2011). However, it is difficult to draw valid conclusions due to the limited number of patients. Therefore, we compared the HBV DNA, cccDNA, total RNA, and pgRNA levels among T, NT, and FNT samples in the 46 HBsAg-positive patients. Four HBV markers do not trend consistently across T, NT, and FNT compartment. As shown in Figure 1A, HBV DNA level was highest in T samples and decreased in NT samples (T vs. NT, *p* = 0.02) and FNT samples (T vs. FNT, *p* = 0.001) in turn, the differences among three tissues were all reaching statistical significance (NT vs. FNT, *p* = 0.03). However, no significant difference of HBV cccDNA level was found among T, NT, and FNT samples (Figure 1B). These results suggest that HBV DNA is not a good marker to reflect viral replication in tumor tissues.

The pgRNA expression in T samples were significantly lower than in NT samples (*p* = 0.02) (Figure 1C). The total RNA expression level in T samples were significantly lower than in NT samples (*p* < 0.001) and FNT samples (*p* < 0.001) (Figure 1D). The lowest expression level of pgRNA and total RNA were found in T samples, suggesting that HBV transcription in T samples is extremely limited. The similar results were shown in IHC analyses of HBcAg expression. HBcAg expressed more frequently and highly in NT and FNT samples than in T samples (Figure 1E).

**FIGURE 2:**
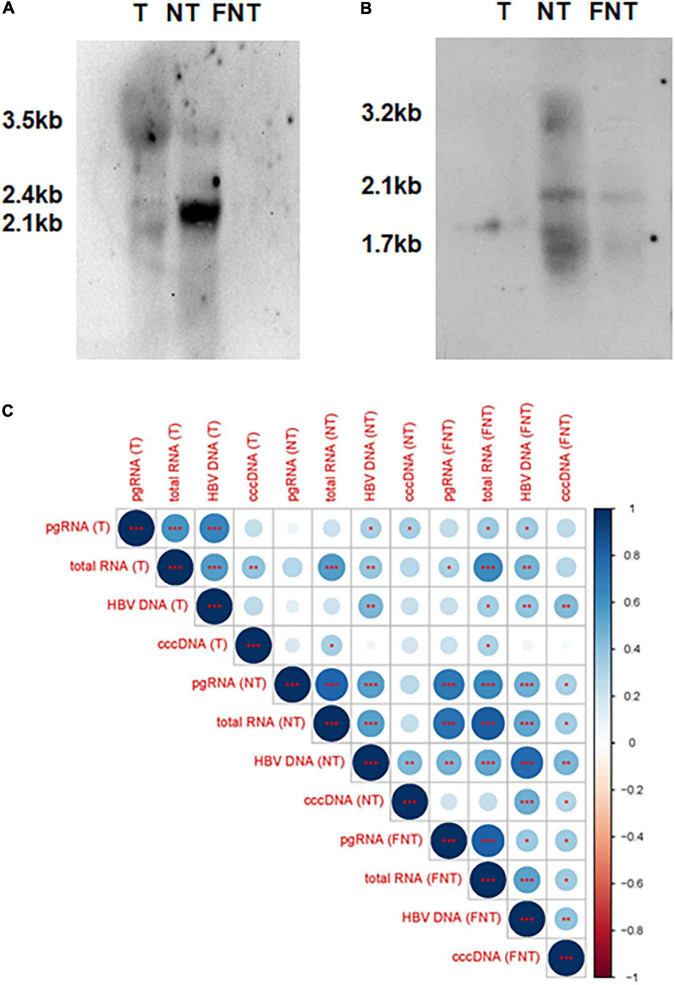
Comparisons and correlations of HBV markers among T, NT, and FNT compartments. **(A)** Northern blot of HBV RNAs marker. **(B)** Southern blot of HBV DNA markers. **(C)** Correlations between HBV markers (DNA, cccDNA, total RNA, and pgRNA) within each compartment and serum HBV DNA. The intersection of the column and row indicates the correlation between the two variables, the color of the circle indicates positive and negative correlation (blue for positive correlation, red for negative correlation), and the diameter of the circle indicates the size of the correlation coefficient. Statistical analysis was performed by Spearman rank correlation test *p < 0.05; ^**^p < 0.01; ^***^p < 0.001. T, tumor; NT, adjacent non-tumor; FNT, distal non-tumor.

To further demonstrate that the expression of these transcripts and proteins are not only derived from integrated DNA, the southern and northern blotting experiments were done on five paired T, NT, and FNT samples. Only one T sample with low cccDNA detection (24 copies/100 ng) showed the pgRNA from signal (Figure 2A) and the cccDNA signal was detected in paired NT samples (Figure 2B) with cccDNA detection of 651 copies/100 ng, which suggests low cccDNA amounts of HBV could also support the production of pgRNA in the tumor environment.

Then, the correlations among the four HBV markers and serum HBV DNA were analyzed by Spearman correlation analyses as shown in Figure 2C. The result revealed a significant positive correlation between any two of the HBV DNA, pgRNA, and total RNA in T, NT, and FNT samples. The correlation between pgRNA and cccDNA was only found in FNT samples, suggesting that the limitation of pgRNA production was independent of the level of cccDNA. These implies that the production of pgRNA was disordered in both T and NT samples relative to the FNT samples. On the other hand, the positive significant correlation with serum HBV-DNA was found only for total RNA and cccDNA in FNT samples, which suggested the difference between HBV replication in the tissue and in the clinical detection. It also highlights that the distal non-tumor tissues are most correlated with serological detection.

### Hepatitis B Virus Virological Changes Among T, NT, and FNT Samples

To characterize the episomal HBV DNA, we amplified viral RT and S in T, NT, and FNT samples of 46 HBsAg-positive patients using a nested PCR and performed phylogenetic tree analysis. We successfully acquired 88.4% (122/138; 40 in T, 40 in NT, and 42 in FNT) of RT sequences, 92% (127/138; 44 in T, 41 in NT, and 42 in FNT) of S sequences, and only 53.6% (74/138; 24 in T, 26 in NT and 24 in FNT) of X sequences.

**FIGURE 3:**
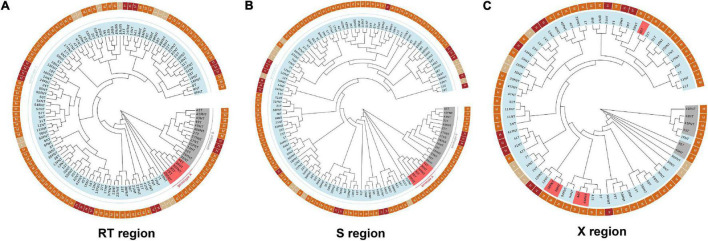
Phylogenetic trees based on **(A)** RT, **(B)** S, and **(C)** X region of HBV in different patients of all tissues. In inner circle, the light blue color means genotype C, the gray color means genotype B, and the red means genotype A. In the outer circle, beige means the patients with low differentiated cancer cell. Orange means the patients with moderate differentiated cancer cells. Deep red means high differentiation group. RT, reverse transcriptase; T, tumor; NT, adjacent non-tumor; FNT, distal non-tumor.

As shown in Figure 3, phylogenetic trees analysis showed that most patients of the evolutionary pattern of RT sequences were relatively consistent among T (Figure 3A), NT (Figure 3B), and FNT (Figure 3C) tissues except four patients, indicating the similar HBV sequences between T, NT, and FNT tissues. However, 11 (#8, #15, #16, #18, #25, #28, 33#, #49, #50, #54, and #56) patients (23%, 11/46) who showed S sequences were clustered far away from T to NT and FNT tissues. Interestingly, Patient (#25) showed genotype A in T and FNT samples, but genotype C in NT samples. This is evident that HBV DNA can be compartmentalized in HCC samples at the sequences and genotype level, indicating that HBV evolves differently in T, NT, and FNT tissues of the same individual. Furthermore, we carried out an inverse PCR to amplify the integrated HBV RT including S gene in 46 HBsAg-positive HCC patients. We successfully detected the RT sequence in T samples of 16 patients (34.7%; 16/46), in NT samples of 15 patients (32.6%, 15/46), and in FNT samples of 16 patients (34.7%, 16/46) (Table 2). In addition, no bias in the distribution of integrated fragments in human genome chromosomes across T, NT, and FNT samples was found (Table 3).

**TABLE 2:** The HBV RT sequence integration among T, NT, and FNT.

Tissue	Samples	Integration numbers	Integrated rate (%)	*p*-value
T	46	16	34.7	1.0
NT	46	15	32.6	
FNT	46	16	34.7	

*T, tumor; NT, adjacent non-tumor; FNT, distal non-tumor.*

**TABLE 3:** The description of HBV RT integration in tumor samples.

Sample	Position of junctions in human genome	Integrated gene	Differentiation degree of sample
1T	Alphoid-like repetitive region begins at Chr17:25645890	Alphoid-like repetitive	Middle differentiation
1T	3′UTR of ABCG2 begins at Chr4:88107421	ABCG2	Middle differentiation
1T	Alpha-satellite repeat region begins at Chr17:24584495	Alpha-satelite repeat	Middle differentiation
1NT	Unannotated region begins at Chr8: 70552	Unknown	Middle differentiation
1NT	Unannotated region begins at Chr16:27210279	Unknown	Middle differentiation
1FNT	3′UTR of ABCG2 begins at Chr4:88107421	ABCG2	Middle differentiation
1FNT	Unannotated region begins at Chr18:6195357	Unknown	Middle differentiation
5NT	Unannotated region begins at Chr4:200064	Unknown	Middle differentiation
5FNT	Unannotated region begins at Chr11:61186	Unknown	Middle differentiation
7T	Unannotated region begins at Chr14:117799	Unknown	Low differentiation
7T	Unannotated region begins at Chr5:32508	Unknown	Low differentiation
8T	Alpha-satelite repeat region begins at Chr15:16712928	Alpha-satelite repeat	Middle differentiation
8T	Unannotated region begins at Chr16:34813818	Unknown	Middle differentiation
8NT	Unannotated region begins at Chr20:35864791	Unknown	Middle differentiation
8FNT	INTRON of FN1 begins at Chr2:215401837	FN1	Middle differentiation
8FNT	Unannotated region begins at Chr18:49818177	Unknown	Middle differentiation
9T	Unannotated region begins at Chr5:137560	RGS6	Middle differentiation
9FNT	INTRON of RGS6 begins at Chr14:72006903	Unknown	Middle differentiation
10T	Unannotated region begins at Chr19:51725260	Unknown	Middle differentiation
11T	INTRON of WAC begins at Chr10:28559310	WAC	Middle differentiation
11NT	INTRON of FN1 begins at Chr2:215412233	FN1	Middle differentiation
12NT	Unannotated region begins at Chr5:119778	Unknown	High differentiation
13T	INTRON of ST3GAL4 begins at Chr11:126442216	ST3GAL4	Middle differentiation
13FNT	Unannotated region begins at Chr2:31050	Unknown	Middle differentiation
19T	INTRON of FAT4 begins at Chr4:125363717	FAT4	Middle differentiation
19T	Unannotated region begins at Chr18:22131992	Unknown	Middle differentiation
19NT	EXON of SPOCK1 begins at Chr5:137313301	Unknown	Middle differentiation
19FNT	EXON of SPOCK1 begins at Chr5:137313301	Unknown	Middle differentiation
22NT	Unannotated region begins at Chr7:25563	Unknown	Middle differentiation
23T	Unannotated region begins at Chr8:59703	Unknown	Middle differentiation
26FNT	3′UTR of ADD1 begins at Chr4:2846896	ADD1	Middle differentiation
27NT	Unannotated region	Unknown	High differentiation
27FNT	INTRON of CEP270 begins at Chr1:243152649	CEP270	High differentiation
27FNT	Unannotated region begins at Chr12:29796	Unknown	High differentiation
33NT	INTRON of ALDH18A1 begins at Chr10:95651615	ALDH18A1	Middle differentiation
33FNT	Unannotated region begins at Chr17:36911338	Unknown	Middle differentiation
36T	Unannotated region begins at Chr11:36389	Unknown	Middle differentiation
36T	Unannotated region begins at Chr15:69200895	Unknown	Middle differentiation
37T	Unannotated region begins at Chr15:98132987	Unknown	Middle differentiation
37NT	INTRON of SPARC begins at Chr5:137390135	SRARC	Middle differentiation
41T	Unannotated region	Unknown	High differentiation
43T	Alpha-satelite repeat region begins at Chr21:11290266	Alpha-satelite repeat	Middle differentiation
47T	Alpha-satelite repeat region begins at Chr18:15964676	Alpha-satelite repeat	Middle differentiation
47FNT	Unannotated region begins at Chr16:96310738	Unknown	Middle differentiation
49FNT	Unannotated region begins at Chr11:167793	Unknown	Middle differentiation
49FNT	Unannotated region begins at Chr10:105089	Unknown	Middle differentiation
57NT	Unannotated region begins at Chr3:17652	Unknown	Middle differentiation
58T	Unannotated region begins at Chr10:9188	Unknown	Middle differentiation
58NT	INTRON of EWSR1 begins at Chr22:29295328	EWSR1	Middle differentiation
58NT	INTRON of ESRRG begins at Chr1:216909872	ESRRG	Middle differentiation
59FNT	Unannotated region begins at Chr5:480	Unknown	Middle differentiation

*RT, reverse transcriptase; UTR, untranslated region; H, high; M, middle; L, low.*

### Comparison of Cancer Stem Cell Related Gene Expressions Among T, NT, and FNT

**FIGURE 4:**
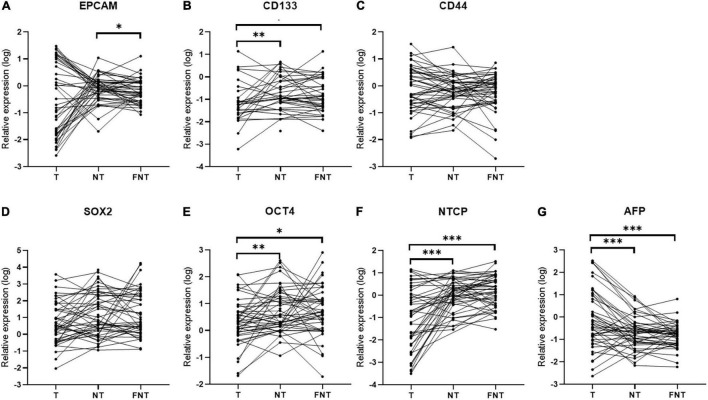
Cancer-related markers expression in T, NT, and FNT samples. Dot plots of **(A)** EPCAM, **(B)** CD133, **(C)** CD44, **(D)** SOX2, **(E)** OCT4, **(F)** NTCP, and **(G)** AFP. Statistical analysis was performed by Wilcoxon signed rank test. *p < 0.05; ^**^p < 0.01; ^***^p < 0.001. EPCAM, epithelial cell adhesion; AFP, alpha fetoprotein; OCT4, octamer-binding transcription factor 4; NTCP, sodium taurocholate cotransporting polypeptide.

Then we compared these markers (EPCAM, CD133, CD44, SOX2, OCT4, NTCP, and AFP) expression among T, NT, and FNT samples. As shown in Figure 4, the expression of EPCAM was higher only in NT than FNT samples (*p* = 0.02) (Figure 4A). The expression of CD133 (T vs. NT, *p* < 0.01; T vs. FNT, *p* = 0.01) (Figure 4B), OCT4 (T vs. NT, *p* < 0.01; T vs. FNT, *p* = 0.03) (Figure 4E), and NTCP (T vs. NT, *p* < 0.001; T vs. FNT, *p* < 0.001) in T samples was significantly lower than NT and FNT samples (Figure 4F). The expression of AFP was higher in T samples than NT (*p* < 0.001) and FNT (*p* < 0.001) samples (Figure 4G).

### The Relationship of Hepatitis B Virus Markers and Cancer Stem Cell-Related Marker Expressions

**FIGURE 5:**
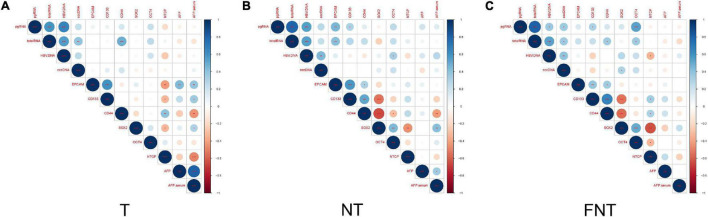
Correlation of CSCs markers and HBV related markers. Correlation between HBV markers (DNA, cccDNA, total RNA, and pgRNA) within T **(A)**, NT **(B)**, and FNT **(C)** groups. The intersection of the column and row indicates the correlation between the two variables, the color of the circle indicates positive and negative correlation (blue for positive correlation, red for negative correlation), and the diameter of the circle indicates the size of the correlation coefficient. Statistical analysis was performed by Spearman rank correlation test *p < 0.05; **p < 0.01; ***p < 0.001.

To investigate the relationship of HBV and CSC-related markers, we did correlation analysis of these markers in T, NT, and FNT samples. In the T samples, we did not find any other significant correlation between HBV and CSC-related markers (Figure 5), except for the significant positive correlation between total RNA and CD44 (*p* < 0.001) (Figures 5B,C).

In contrast, the levels of HBV pgRNA were positively correlated to EPCAM (*p* < 0.01) and CD133 (*p* < 0.05), and total RNA expression levels were positively correlated to EPCAM (*p* < 0.01) in NT samples. At the same time, HBV DNA had also shown positive correlations with OCT4 (*p* < 0.05) in NT samples (Figure 5B).

The pattern of correlations in FNT samples also differed from NT samples (Figure 5C), the levels of HBV pgRNA were positively correlated to CD133 (*p* < 0.05) and OCT4 (*p* < 0.001), and total RNA levels were positively correlated to CD44 (*p* < 0.05) and OCT4 (*p* < 0.05). HBV cccDNA and serum HBV DNA were positively correlated to SOX2 (Figure 5C). Other than that, CSC markers were not found to be significantly correlated with the other HBV markers in any tissues. Similar results were shown in IHC analyses of OCT4. The spatial location of OCT4 expression was also consistent in HBcAg expression in FNT samples (Supplementary Figure 1).

These results suggested that HBV markers showed positive correlations with CSCs markers in NT and FNT samples.

### Clinical Correlations

**FIGURE 6:**
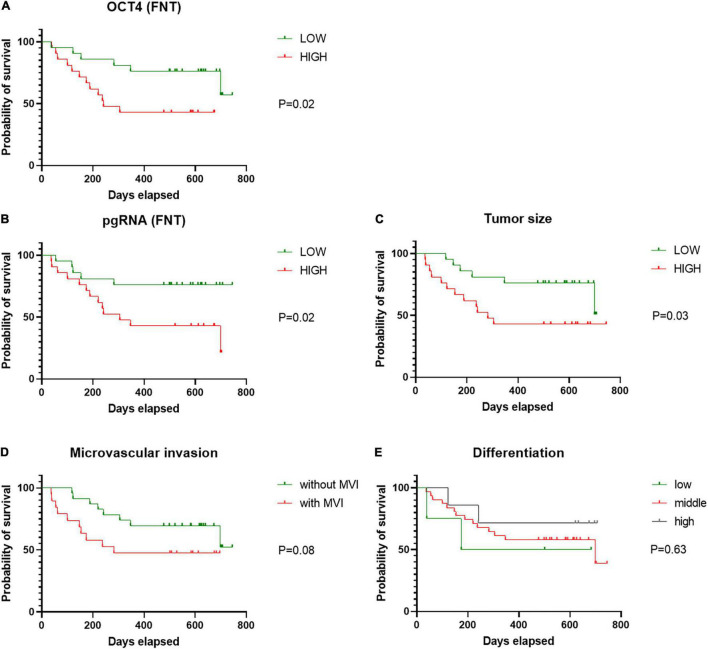
Cancer recurrence of patients according to pgRNA (FNT). Cancer recurrence of patients according to **(A)** OCT4 (FNT), **(B)** pgRNA (FNT), **(C)** tumor size, **(D)** mvi, and **(E)** differentiation. Statistical analysis was performed by log-rank test. FNT, distal non-tumor; mvi, microvascular invasion.

Continuous variables (tumor size, pgRNA, and OCT4 level) were divided into binary categorical variables by median. Under survival analysis, patient’s cancer recurrence was significantly lower in those patients with low level of pgRNA in FNT samples (Figure 6A), low level of OCT4 in FNT samples (Figure 6B), and the tumor size smaller than 5.5 cm (Figure 6C), but not other factors such as no tumorous vascular invasion (Figure 6D) and differentiation (Figure 6E), indicating that pgRNA and OCT4 in FNT could be a significant predictive factor for cancer recurrence. Meanwhile, to address the concern that the antiviral therapy may be the real reason leading to different prognosis in HCC patients, we analyzed viral and CSCs markers in antiviral treatment patients compared to those patients without treatment. As shown in Supplementary Figure 2, there were no significant differences of viral markers between patients with and without viral treatment. However, OCT4 in T (Supplementary Figure 3A) and EPCAM in NT (Supplementary Figure 3B) and FNT (Supplementary Figure 3C) of patients with antiviral treatment showed significant lower levels than those patients without any antiviral treatment. In addition, we also performed survival analysis for the patients with or without antiviral therapy in the same way. Patients with antiviral treatment could be a predictive factor for cancer recurrence (Supplementary Figure 4). The results still indicated that pgRNA in FNT could also be a significant predictive factor in patients without antiviral therapy, and OCT4 expression in FNT showed the same trend in predicting recurrence although there was no significant difference (*p* = 0.06) (Supplementary Figure 5). Moreover, we performed multivariate cox regression analysis for pgRNA (FNT), in combination with OCT (FNT) microvascular invasion (mvi), differentiation, tumor size, and antiviral therapy. In multivariate cox regression analysis for pgRNA (HR = 4.541, *p* = 0.033), tumor size (HR = 5.423, *p* = 0.008), antiviral therapy (HR = 7.945, *p* = 0.004), and OCT4 (HR = 3.456, *p* = 0.072) were in the equation (Table 4), which means the high pgRNA level group was the risk factor for recurrence. We also used the data annotated with HBV antigen from TCGA liver cancer for supplementary testimony. Although the number of paired annotation samples (*N* = 22) is small, OCT4 also showed a trend of predictive value in adjacent normal tissues (*p* = 0.07) relative to tumor tissues (*p* = 0.31) in survival analysis (Supplementary Figure 6).

**TABLE 4:** Variables in the equation of multivariate cox regression analysis for pgRNA.

	B	SE	Wald	*df*	Sig	Exp(B)
PgRNA (FNT)	1.513	0.708	4.563	1	0.033	4.541
OCT4 (FNT)	1.240	0.688	3.246	1	0.072	3.456
Anti-viral treatment (AVT)	2.073	0.711	8.486	1	0.004	7.945
Tumor size	1.691	0.634	7.110	1	0.008	5.423

These data suggest that HBV replication marker of pgRNA correlates to CSCs markers (OCT4) with the trend of predictive value in adjacent normal tissues.

## Discussion

In the present study, our results showed HCC in HBsAg-positive patients can still present low HBV replication with low level of pgRNA and different sequences between T and NT samples in some patients. In addition, HBV markers were more frequently significantly correlated with CSC markers in non-tumor samples. The level of pgRNA and OCT4 in FNT samples are predictive markers of HCC recurrence after liver cancer resection therapy.

The pgRNA level reflects HBV transcription from cccDNA, which is the template for HBV transcription (Li et al., 2017). The pgRNA is the pivotal marker reflecting HBV replication. We used multiple methods to determine the reliability of our HBV marker quantification, including the use of Northern blot and Southern blot to confirm the presence of pgRNA and cccDNA, and IHC for HBcAg to detect protein levels. Our data show the expression level of total RNA and pgRNA were lower in tumor samples than non-tumor samples; this is consistent with previous data (Halgand et al., 2018). However, the levels of HBV DNA and cccDNA were not low in T samples. On the contrary, HBV DNA has the highest load in the T samples. It might result from the accumulation of integrated fragments due to the perpetual amplification of tumor cells. It could not be confirmed by Inverse PCR, because integration detection assays could only detect different integrations and cannot quantify integration fragments. Meanwhile, our data also reproduce that different genotypes may exist in different tissues as previous studies reported (Figure 3; Amaddeo et al., 2015).

Cancer stem cells are thought to be responsible for generating a heterogeneous tumor lesion and contributing to treatment resistance, tumor relapse, and metastasis (Zheng et al., 2018). Several previous studies have shown that HBV infection can promote cancer development by affecting the expression of liver CSCs markers. Mani et al. (2016) reported that HBV infection up-regulates EPCAM expression and induces CSCs like gene signature. Park et al. (2020) showed that HBV replication suppresses HNF4α, which contributes to tumor cell proliferation. In addition, Liu et al. (2017) showed that HBV preS1 promotes the appearance and self-renewal of CSCs to facilitate HCC. While most of the previous studies were based on *in vitro* experiments, our work presented the relationship between the levels of HBV markers and liver CSCs markers in the real world. However, the levels of CSCs markers were not highly expressed in T samples. Instead, CD133 and OCT4 were more highly expressed in NT and FNT samples than in T samples (Figure 4). Current research suggested liver CSCs might originate from liver stem/progenitor cells, mature parenchymal cells, and differentiated liver cancer *via* transformation and dedifferentiation, respectively (Nio et al., 2017). These high expression levels of the CSC markers in NT tissues is consistent with the HBV transcript (total RNA and pgRNA) expression profile. The correlation analysis also confirmed that CSC markers significantly correlated to HBV markers in NT and FNT samples. In particular, pgRNA level in FNT samples positively correlated to OCT4 (Figures 4, 5). To obtain more evidence, immunohistochemistry was performed on serial sections of the same samples. The results showed that OCT4 expression levels correlated to HBcAg expression (Supplementary Figure 1), which exhibit a similar trend for the correlation in mRNA expression. Moreover, survival analysis showed that OCT4 and pgRNA in FNT samples were the factor of predictive value of HCC recurrence after resection therapy (Figure 6) and the expression of pgRNA in FNT samples was the risk factor for recurrence in multivariate cox regression analysis (Table 4). These data support the theory that HBV replication can induce CSCs transform and HCC recurrence through upregulating the expression of CSCs marker OCT4.

In evaluating the recurrence after resection therapy, it is obvious that studying the status of non-tumor tissues is of greater practical value, as the resected tumor tissue cannot continue to affect the patients. In our study, we included FNT tissues for analysis in addition to using adjacent non-tumor tissues as controls. Our results showed that most of the characteristics of the NT samples were similar to those of the FNT samples, including the presence of significant differences from the T samples and significant correlation among CSC markers that did not appear in the T samples. However, two key features suggest important differences between NT and FNT samples. One was that the correlation between pgRNA and cccDNA appeared only in FNT samples (Figure 2C). As we know, HBV pgRNA expression in normal cells is positively correlated to cccDNA. The pgRNA and cccDNA were no longer significantly correlated in tumor tissues because viral replication is restricted. In our results, the significant positive correlation between pgRNA and cccDNA was restored only in FNT samples (Figure 2C), indicating that viral replication in NT samples was also affected. The other feature was that the AFP produced in NT samples rather than FNT samples appeared significantly correlated with serum AFP (Figure 5). The liver cancer cells can regain the ability to synthesize AFP, which was lost in mature hepatocytes, and increase the AFP content in serum (Wang and Wang, 2018). Therefore, the high correlation between the level of AFP in NT samples and those in serum demonstrated the abnormality of NT samples compared to FNT samples. Thus, our data suggested that the adjacent non-tumor tissues, which already differs from normal hepatocytes in HBV replication and oncogene expression, was not the best healthy control for the tumor tissues. The status of distal non-tumor tissues should be more closely monitored in predicting recurrence, as they may be more reflective of the true normal hepatocytes. In addition, these data also suggest that antiviral therapy would be an important way to prevent the recurrence.

We recognized some limitations in this study. Firstly, the total number of enrolled patients was small, and there were very few HBsAg-negative patients, so the comparative analysis would be subject to some sampling error. Secondly, we did not obtain paired blood samples to validate the predictive value of the content of pgRNA in serum.

## Conclusion

In conclusion, our data suggested that HBV replication may induce HCC recurrence by upregulating CSC markers of OCT4 expressions in non-tumor tissues. The pgRNA in normal tissues might be helpful to predict the recurrence of HCC patient after treatment. Hence, HBV infection caution is warranted when treating HCC patients, and further studies should focus on the mechanism of HBV-induced CSCs.

## Data Availability Statement

The original contributions presented in the study are included in the article/Supplementary Material, further inquiries can be directed to the corresponding authors.

## Ethics Statement

The studies involving human participants were reviewed and approved by the Ethics Committees of the Medical Center (No. S2018-111-01). The patients/participants provided their written informed consent to participate in this study.

## Author Contributions

KX, HZ, and SL designed research. YXi was responsible for the final statistics of the detection data. JC was responsible for matching the final clinical data. YXi and CZ collected the samples. YXi, CZ, ZZ, GO, JC, JS, ZL, YL, YXu, WZ, and JD performed the experiments. KX, HZ, TL, SL, and JL analyzed the data. KX, HZ, SL, and TL wrote and revised the manuscript. All authors provided critical review of the manuscript and approved the submitted version.

## Conflict of Interest

The authors declare that the research was conducted in the absence of any commercial or financial relationships that could be construed as a potential conflict of interest.

## Publisher’s Note

All claims expressed in this article are solely those of the authors and do not necessarily represent those of their affiliated organizations, or those of the publisher, the editors and the reviewers. Any product that may be evaluated in this article, or claim that may be made by its manufacturer, is not guaranteed or endorsed by the publisher.

## Footnotes

1
http://xena.ucsc.edu

## Abbreviations

HCC: hepatocellular carcinoma
HBV: hepatitis B virus
cccDNA: covalently closed circular DNA
pgRNA: pregenomic RNA
CSC: cancer stem cell
EPCAM: epithelial cell adhesion
OCT4: octamer-binding transcription factor 4
NTCP: sodium taurocholate cotransporting polypeptide
HNF4α: hepatocyte nuclear factor 4α
T: tumor
NT: adjacent non-tumor
FNT: far distal non-tumor
RPS11: ribosomal protein S11
RT: reverse transcriptase
L: low
M: middle
H: high
P: positive
N: negative.
