# Supplementary material for: Hepatitis B Virus Pregenomic RNA Reflecting Viral Replication in Distal Non-tumor Tissues as a Determinant of the Stemness and Recurrence of Hepatocellular Carcinoma
Source: Front Microbiol. 2022 Apr 7;13:830741. doi: 10.3389/fmicb.2022.830741 (PMC9021960; doi:10.3389/fmicb.2022.830741)
Supplement: Supplementary file 1 [file Data_Sheet_1.docx]

Supplementary Material

# Supplementary Tables

**Table S1. Primers for the experiments**

| Primers | Primer sequence (5’-3’) | Function |
| --- | --- | --- |
| BJMU-00054 | GAGTGTGGATTCGCACTCC | HBV pgRNA |
| BJMU-00055 | GAGGCGAGGGAGTTCTTCT |  |
| BJMU-00056 | AAGCCACCCAAGGCACAG | HBV total RNA |
| BJMU-00057 | GCACCAGCACCATGCAAC |  |
| BJMU-00058 | CCGTCTGTGCCTTCTCATCTG | HBV DNA |
| BJMU-00059 | AGTCCAAGAGTCCTCTTATGTAAGACCTT |  |
| BJMU-00060 | 56-FAM/CCGTGTGCA/ZEN/CTTCGCTTC ACCTCTGC/3IABkFQ | Probe for HBV DNA |
| BJMU-00061 | GCCGAGACTATCTGCACTAC | RPS11 |
| BJMU-00062 | ATGTCCAGCCTCAGAACTTC |  |
| BJMU-00063 | TGCACTTCGCTTCACCT | HBV cccDNA |
| BJMU-00064 | AGGGGCATTTGGTGGTC |  |
| BJMU-00182 | AATCGTCAATGCCAGTGTACTT | EPCAM |
| BJMU-00183 | TCTCATCGCAGTCAGGATCATAA |  |
| BJMU-00188 | CGGACACCATGGACAAGTTT | CD44 |
| BJMU-00189 | GAAAGCCTTGCAGAGGTCAG |  |
| BJMU-00190 | GGACCCATTGGCATTCTC | CD133 |
| BJMU-00191 | CAGGACACAGCATAGAATAATC |  |
| BJMU-00086 | GCCATTTGTTCAGTGGTTCGTAG | Inverse PCR (first run) |
| BJMU-00148 | AGGCCCACTCCCATAGGAATC |  |
| BJMU-00087 | AGGGCTTTCCCCCACTGTTT | Inverse PCR (second run) |
| BJMU-00143 | TGAGAGAAGTCCACCACGAGTCTAG |  |
| P2 | CTAGGAGTTCCGCAGTATGGAT | HBV RT (first run) |
| P5 | GTGGCTCCAGTTCMGGAACAGT |  |
| P3 | CTCCAGTTCCGGAACAGT | HBV RT (second run) |
| P4 | GCAGAGGAGCCACAAAGG |  |
| BJMU-00207 | TGCCGATCCATACTGCGGAA | HBx |
| BJMU-00208 | ACCCCAACACAGGATAGCTTG |  |

RPS11, ribosomal protein S11; EPCAM, epithelial cell adhesion molecule; RT, reverse transcriptase; pgRNA, pregenomic RNA.

**Table S2. Variables not in the equation of multivariate cox regression analysis of pgRNA in HBsAg-positive patients for recurrence.**

|  | B | df | Sig |
| --- | --- | --- | --- |
| MVI | 1.456 | 1 | 0.228 |
| Differentiation* | 0.228 | 2 | 0.892 |
| Differentiation (1) | 0.018 | 1 | 0.894 |
| Differentiation (2) | 0.087 | 1 | 0.768 |

* There were three groups in Differentiation variable
