# Supplementary material for: Hepatitis B Virus Pregenomic RNA Reflecting Viral Replication in Distal Non-tumor Tissues as a Determinant of the Stemness and Recurrence of Hepatocellular Carcinoma
Source: Front Microbiol. 2022 Apr 7;13:830741. doi: 10.3389/fmicb.2022.830741 (PMC9021960; doi:10.3389/fmicb.2022.830741)
Supplement: Supplementary file 2 [file Data_Sheet_2.docx]

Supplementary Material

# Supplementary Figure legends:

**Supplementary figure 1.** IHC signal strength analysis using Qupath, progressively increasing from blue to red.

**Supplementary figure 2.** HBV related markers expression in T, NT and FNT from patients with antiviral treatment and without antiviral treatment.

**Supplementary figure 3**. CSCs related markers expression T, NT and FNT from patients with antiviral treatment and without antiviral treatment.

**Supplementary figure 4**. Cancer recurrence of patients according to OCT4 and pgRNA in FNT samples in (A) all patients, and (B) patients without AVT. Statistical analysis was performed by log-rank test.

**Supplementary figure 5.** Survival analyses in paired HBV-related patients’ samples from TCGA Liver Cancer project using UCSC Xena according to OCT4 gene in (A) tumor tissues, and (B)adjacent normal tissues. Statistical analysis was performed by log-rank test.
