# Supplementary figures and images for: Hepatitis B Virus Pregenomic RNA Reflecting Viral Replication in Distal Non-tumor Tissues as a Determinant of the Stemness and Recurrence of Hepatocellular Carcinoma
Source: Front Microbiol. 2022 Apr 7;13:830741. doi: 10.3389/fmicb.2022.830741 (PMC9021960; doi:10.3389/fmicb.2022.830741)

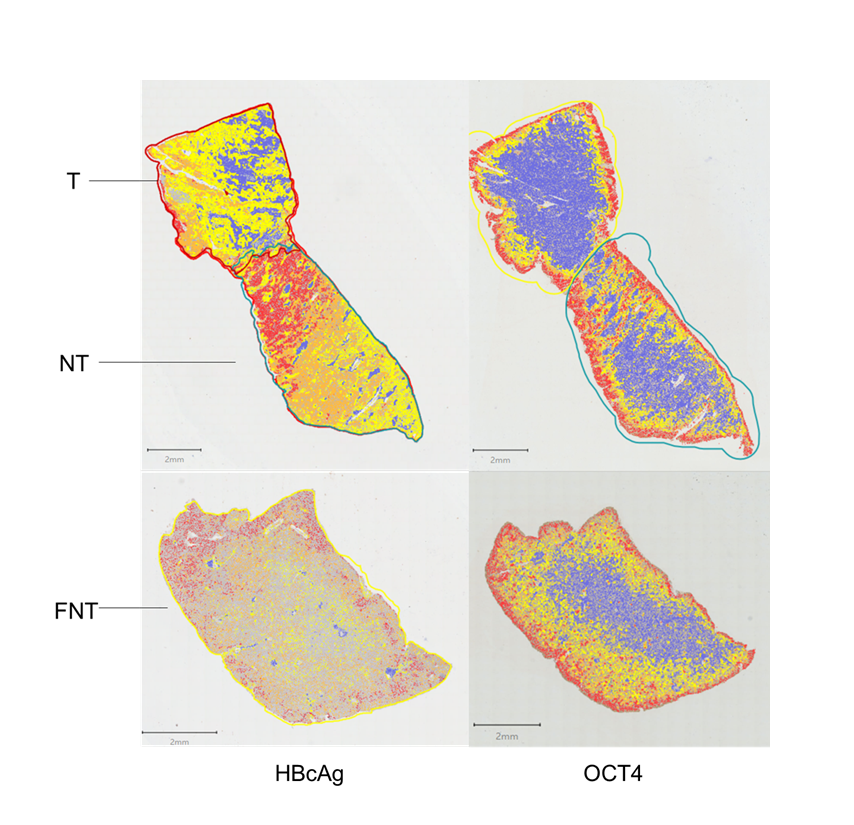

Supplement: Supplementary file 3 [file Image_1.TIF]

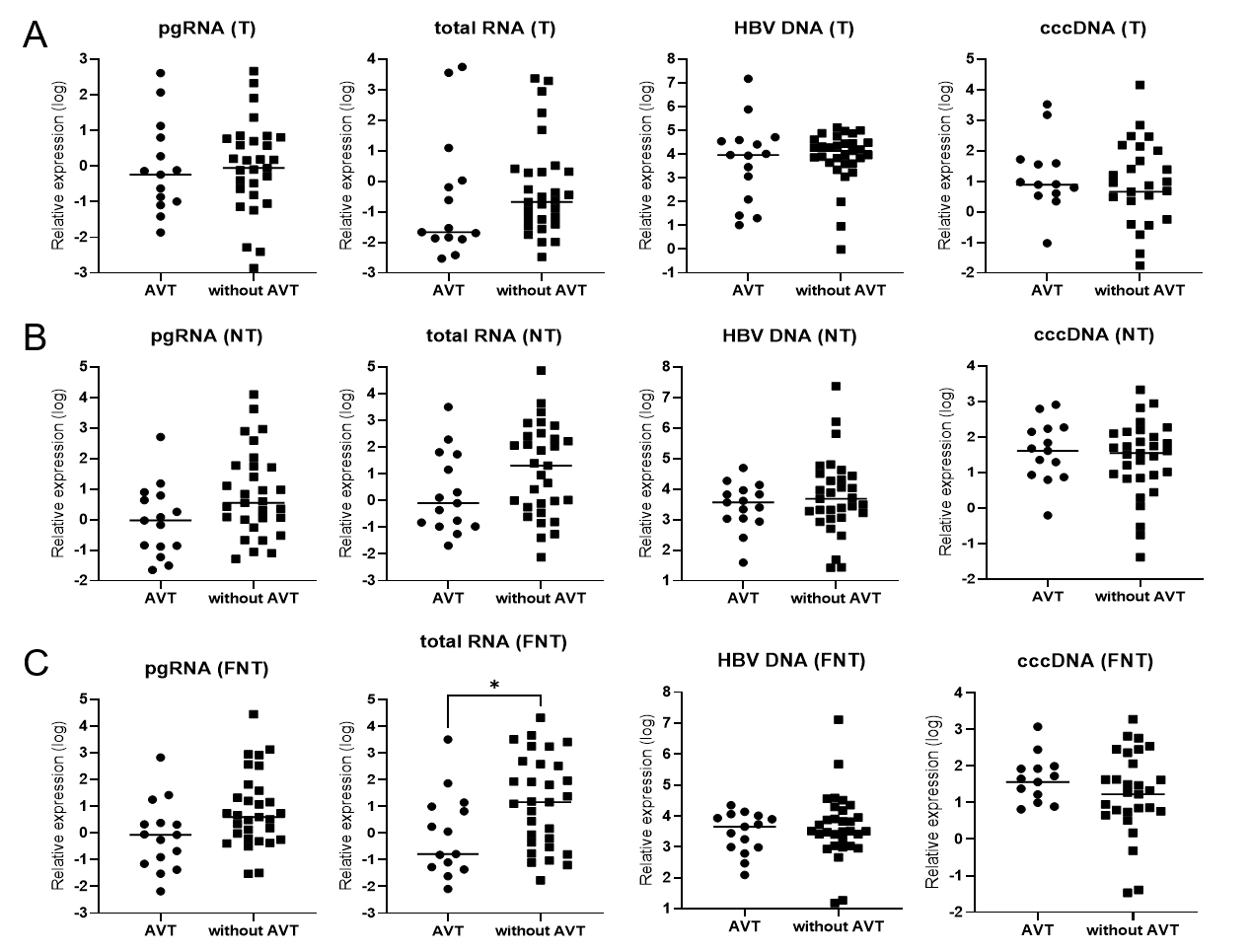

Supplement: Supplementary file 4 [file Image_2.TIF]

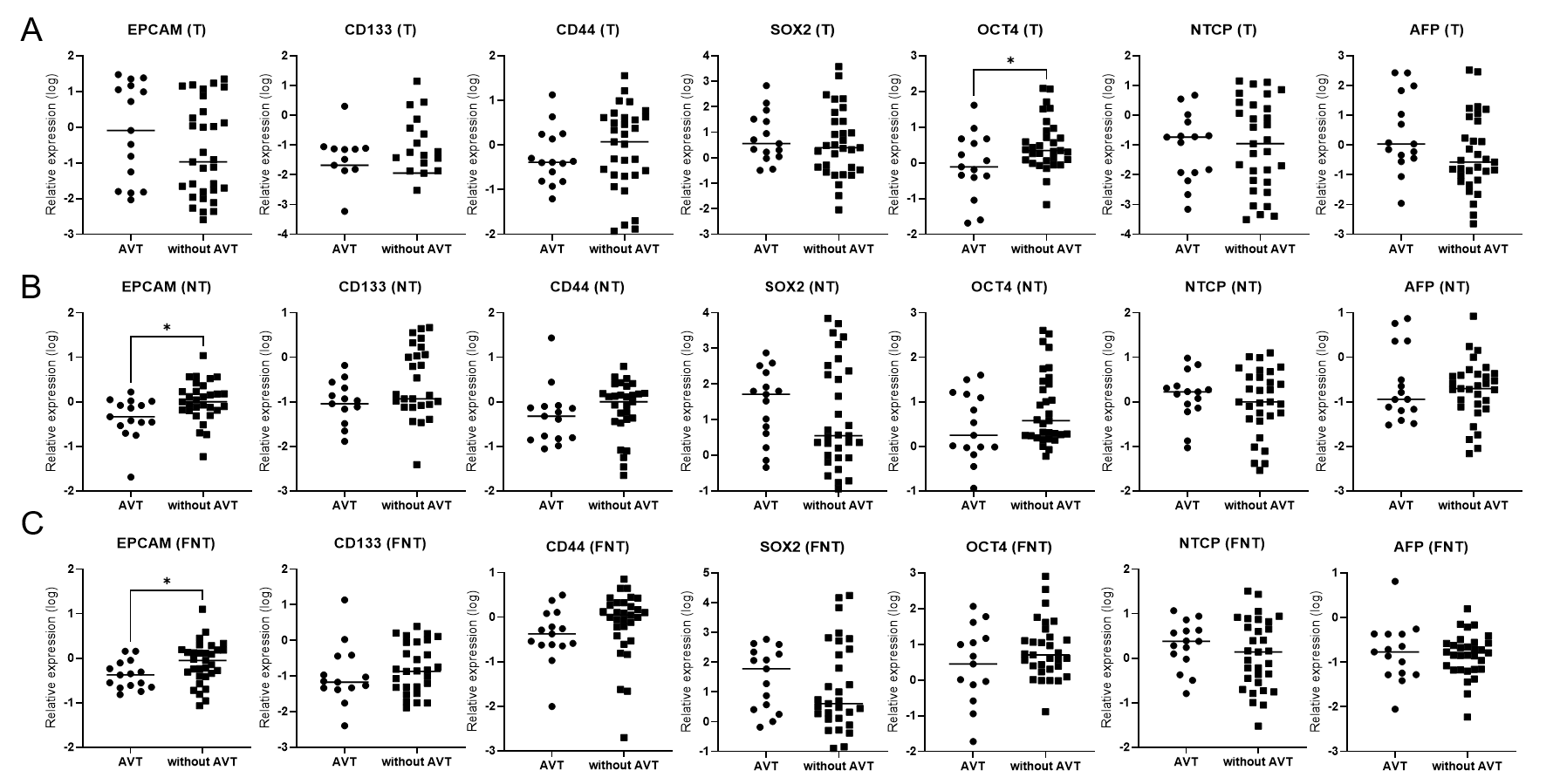

Supplement: Supplementary file 5 [file Image_3.TIF]

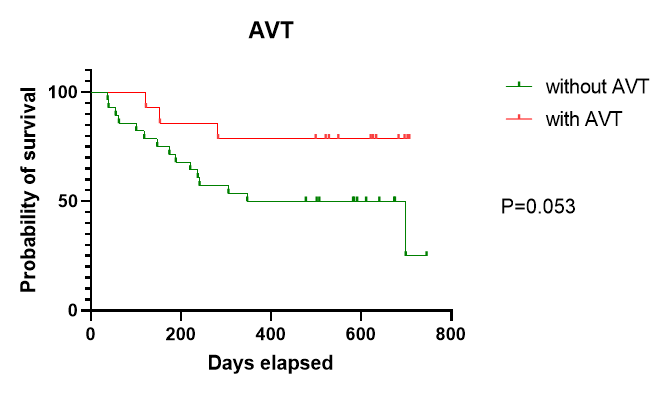

Supplement: Supplementary file 6 [file Image_4.TIF]

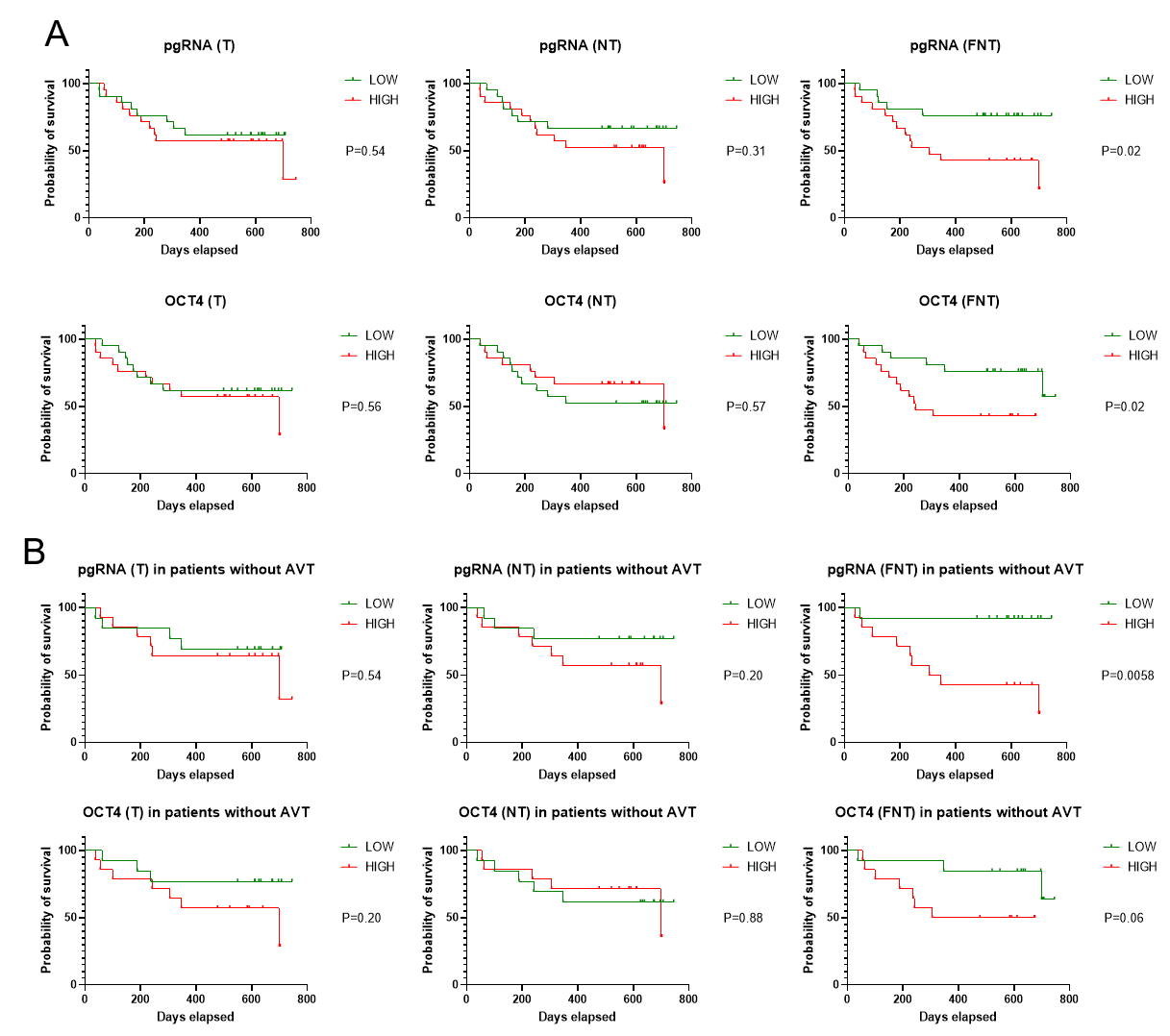

Supplement: Supplementary file 7 [file Image_5.TIF]

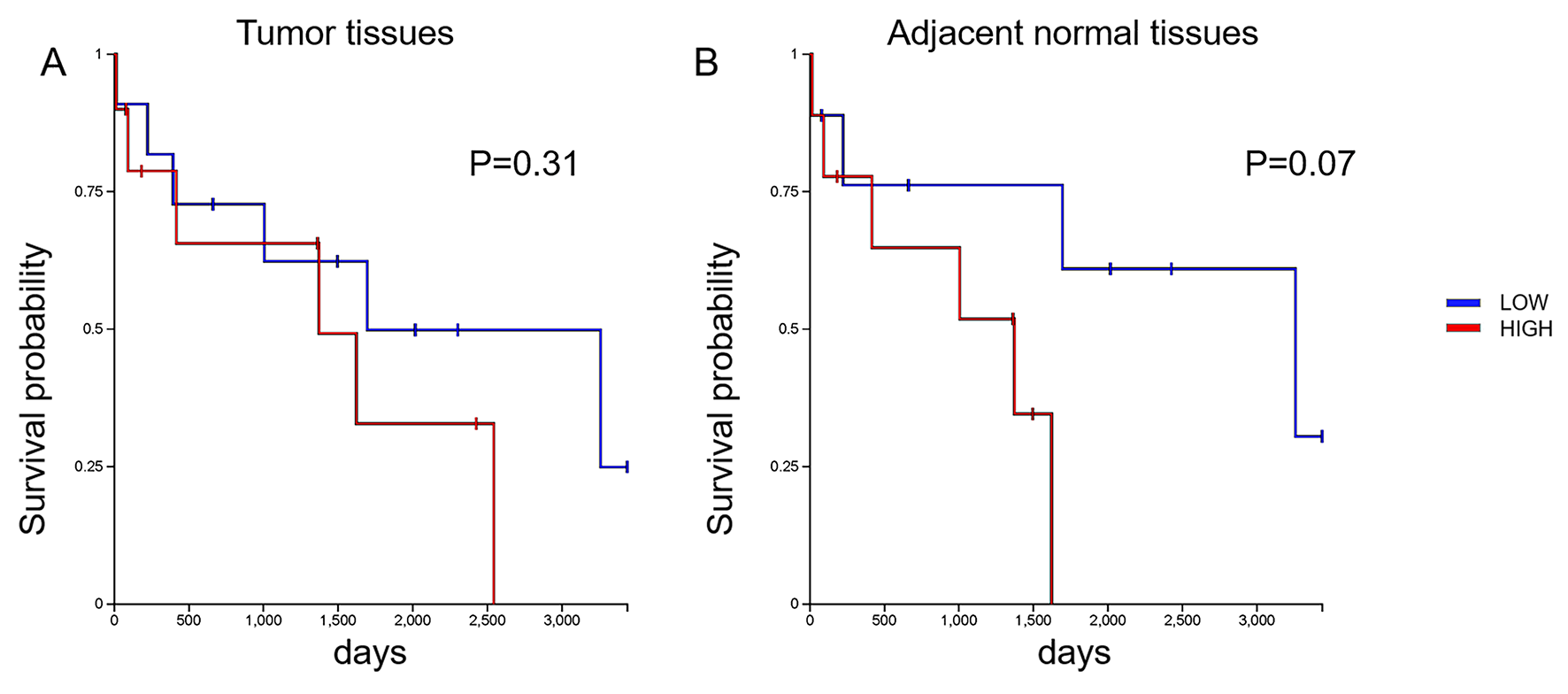

Supplement: Supplementary file 8 [file Image_6.TIF]
